# The Stealth Virus

**DOI:** 10.3201/eid1907.130027

**Published:** 2013-07

**Authors:** Julian W. Tang

**Affiliations:** Alberta Provincial Laboratory for Public Health, Edmonton, Alberta, Canada

**Keywords:** cytomegalovirus, CMV, viruses, stealth, viruses, transplant, HIV, AIDS, pregnant, congenital, immunocompromised

This is a lucid read on a complex virus, cytomegalovirus (CMV), that affects those in all ages of life from the unborn fetus to the elderly pensioner. Griffiths has used one of the many self-publishing, internet-based services to publish and distribute this book via the popular amazon.com and amazon.co.uk websites. All profits from its sale are being donated to the Royal Free Charity, which has supported his research on CMV for many years. 

The book is arranged chronologically; the 18 chapters are headed by a range of years, starting with 1910 (Chapter 1) and ending in 2012 (Chapter 18). An interesting approach is used in the Prologue (dated 180 million years ago), where the virus speaks to the reader in the first person. The virus’s voice summarizes the most important aspects of its virology and its clinical impact on a largely unsuspecting human population. Several important themes are introduced here: 1) the author’s clear views about the relative irrelevance of experiments on mice to the human situation; 2) the missed opportunities that studying CMV earlier could have created by helping the scientific community to understand and more effectively combat other viruses, such as HIV; and 3) how a vaccine approach would be the best way to control this viral pathogen.

The book starts off by discussing other viruses of clinical importance (polio, varicella zoster, rubella, herpes simplex, and HIV), gradually setting the scene for the emergence of CMV. Interspersed between the history of the science and the emerging clinical importance of this virus are patient case histories. At least 1 case is described in each chapter (suitably anonymized with alternative and sometimes memorable names, such as “Stevie Headbanger”), covering the most common clinical CMV scenarios. These include CMV infection and disease in newborns, pregnant women, transplant patients, persons with HIV/AIDS, and other immunocompromised patients. Other cases also demonstrate the virus’ impact on otherwise healthy teenagers studying for exams, retirees caring for their grandchildren, and recovering burn and gunshot victims (in this instance, Pope John Paul II). Such case histories provide clear examples of complications arising from both primary and reactivated CMV infection and disease.

Most (if not all) of the relevant major scientific and medical advances related to CMV are noted, including the development of the various anti-CMV drugs, with due credit given to the investigating teams. Most passionately of all, Griffiths carefully charts gradually changing attitudes to the development of a CMV vaccine.

Curiously, the author writes about his own role in the CMV story in the third person—perhaps to maintain a scientist’s natural objectivity. Notably, in the latter half of the book, he tends to use more technical jargon, which may cause problems for some readers. Yet, this does not necessarily detract from the book’s value; rather, it may provide reassurance to the readers that they are, indeed, in the hands of a master.

The development of a CMV vaccine is now gaining support internationally, thanks in no small part to the efforts of the author. When the vaccine is eventually available, anyone with any doubts about its potential benefits can simply read this little book.

